# What Is New in Metabolic Dysfunction-Associated Steatotic Liver Disease in Lean Individuals: From Bench to Bedside

**DOI:** 10.3390/jcm13010278

**Published:** 2024-01-03

**Authors:** Pojsakorn Danpanichkul, Kanokphong Suparan, Donghee Kim, Karn Wijarnpreecha

**Affiliations:** 1 Immunology Unit, Department of Microbiology, Faculty of Medicine, Chiang Mai University, Chiang Mai 50200, Thailand; 2 Division of Gastroenterology and Hepatology, Stanford University School of Medicine, Stanford, CA 94305, USA; 3 Division of Gastroenterology and Hepatology, Department of Medicine, University of Arizona College of Medicine, Phoenix, AZ 85004, USA; 4 Department of Internal Medicine, Banner University Medical Center, Phoenix, AZ 85006, USA

**Keywords:** metabolic dysfunction-associated steatotic liver disease, liver disease, metabolic syndrome, lean

## Abstract

Metabolic dysfunction-associated steatotic liver disease (MASLD) affects more than 30% of the world’s adult population. While it is associated with obesity and metabolic syndrome, emerging evidence has shown that a substantial number of MASLD patients have a normal body mass index (“lean individuals with MASLD”). In this article, we provide an overview of the definition, epidemiology, pathogenesis, and clinical outcomes associated with lean individuals with MASLD and updates on current management.

## 1. Introduction

Metabolic dysfunction-associated steatotic liver disease (MASLD), previously referred to as nonalcoholic fatty liver disease (NAFLD), affects approximately 30% of adults globally and is the leading cause of liver transplantations in the United States [1,2,3,4,5]. MASLD exhibits a strong association with metabolic syndrome components, including hypertension (HTN), hyperlipidemia (HLD), diabetes mellitus (DM), and obesity [6]. It is noteworthy that although obesity is a well-established risk factor for MASLD, emerging evidence highlights the occurrence of MASLD in individuals without obesity or overweight, leading to the term “MASLD in lean individuals” [7]. Additionally, it should be noted that the term MASLD is more applicable to lean individuals than the previously suggested metabolic dysfunction-associated fatty liver disease (MAFLD) criteria [8].

## 2. Definition of MASLD in Lean Individuals

The classification of individuals into lean (defined as body mass index (BMI) < 25 kg/m^2^ in Western populations and <23 kg/m^2^ in Asians) and non-high BMI (defined as BMI < 30 kg/m^2^ in Western populations and <25 kg/m^2^ in Asians) has been based on BMI [9,10]. This lower threshold for Asians is attributed to the fact that BMI tends to reflect a higher percentage of body fat and related complications compared to Caucasians. While BMI is a convenient tool, it has limitations as it does not provide insights into fat distribution and metabolic status, which are crucial predictors of clinical outcomes [10,11]. Besides BMI, various research endeavors explore body fat distribution through alternative methods such as waist circumference and waist/hip ratio [12]. These measures offer a more accurate reflection of visceral adipose tissue, and waist circumference is included as a criterion for MASLD, though not for lean MASLD. Moreover, techniques like computed tomography and magnetic resonance imaging can assist in quantifying subcutaneous and visceral adipose tissue, although their primary utilization remains within the realm of research [10].

## 3. Epidemiology

Historically, MASLD in lean individuals was primarily seen as an exclusive phenomenon in Asian populations [13]. However, numerous studies have indicated that individuals of other ethnicities are also affected by MASLD in lean form [14]. Globally, it is estimated that around 15% of MASLD cases occur among lean individuals [15]. MASLD in lean individuals is more common in Asians than in other ethnicities [16]. The proportion of MASLD in lean individuals is lowest among Africans [10]. Nonetheless, the available data on MASLD in lean individuals are quite diverse, largely due to variations in fat distribution among different populations and differences in the definition of “lean”. Some studies classify individuals as non-obese, while others use a normal BMI threshold to classify them as lean [17].

## 4. Pathogenesis of MASLD in Lean Individual

Substantial evidence has pointed out that metabolic dysregulation as a consequence of nonmodifiable genetic factors and modifiable lifestyle-related factors plays a crucial part in the pathogenesis of MASLD in most lean individuals [18]. This section unravels those disease-contributing factors, including novel single-nucleotide polymorphisms (SNPs) and cardiovascular (CV) risk factors, along with the clinical course of liver pathology in lean individuals compared with their counterparts. However, it should be noted that alcohol consumption in lean individuals with MASLD is often underreported, which may obscure the understanding of MASLD’s pathogenesis in these patients [19].

Monogenic mutations of genes associated with lipid metabolism or transport, such as aldolase B (*ALDOB*), apolipoprotein B (*APOB*), and alpha/beta hydrolase domain-containing proteins (*ABHDs*), have been reported as causes of MASLD in lean individuals. Still, these mutations are uncommon and account for only a small proportion of lean MASLD [20,21,22]. The development of MASLD in most lean individuals is potentially explained by the collective polygenic effect of SNPs.

Many genes have been associated with the risk of MASLD. Table 1 displays novel MASLD-associated genes and their corresponding SNPs in lean and non-lean individuals with or without MASLD. Most MASLD-associated genes are well documented to contribute to at least a part of lipid metabolism or iron homeostasis [23,24,25,26,27,28,29,30,31,32,33]. In lean individuals, these genes comprise patatin-like phospholipase domain-containing 3 (*PNPLA3*), transmembrane 6 superfamily member 2 (*TM6SF2*), glucokinase regulatory protein (*GCKR*), TBC1 domain family member 1 (*TBC1D1*), human homeostatic iron regulator (*HFE*), solute carrier family 17 member 3 (*SLC17A3*), and fat mass and obesity-associated alpha-ketoglutarate dependent dioxygenase (*FTO*) [23,24,25,26,27,28,29,30,31,32,33].

### 4.1. Polygenic Effect of Single-Nucleotide Polymorphisms on Lean Patients with MASLD

PNPLA3 and TM6SF2 are by far the most widely studied MASLD-associated genes in lean individuals. In the case of PNPLA3 (rs738409; C/G), both homozygous and heterozygous missense variants (GG and CG) may play a role in the development of MASLD as well as its liver-related complications, notably cirrhosis, in both lean and non-lean individuals in an additive manner [23,24,25,26,27,28,29,30]. TM6SF2 (rs58542926; C/T), another missense variant, had inconsistent genotype patterns in driving the pathogenesis of MASLD in lean individuals in different countries [23,24,25,26,29]. For example, homozygosity for the risk allele (TT) was more common in MASLD in lean individuals (4%) than in MASLD in non-lean individuals with MASLD (0.3%) [26]. In contrast, in a UK study, both lean and non-lean patients with MASLD had an increase in the T-allele frequency of the variant relative to individuals without the disease [29]. Yet, in Austria and Hong Kong, the T allele was not associated with MASLD, either in lean or non-lean individuals [24,25]. This could be due to differences in ethnicities, BMI, and other confounding factors such as alcohol consumption. Nevertheless, further study regarding TM6SF2 needs to be conducted to better assess the implications of this variant [34,35]. Some studies unraveled intricate interrelationships between a variant and a CV risk factor. One study of TBC1D1 (rs2279028; A/G) found that a low level of high-density lipoprotein (HDL) may be driven by either a homozygous A or G allele in an overdominant manner [32]. In addition, various intron variants of FTO (rs1421085, T/C; rs3751812, G/T; rs8050136, C/A; and rs9939609, T/A) may be attributed to an increase in the level of low-density lipoprotein (LDL) in a recessive manner [33]. Another interesting finding demonstrated that an increase in the C allele frequency of GCKR (rs1260326; T/C) was associated with a larger waist circumference (WC) in a dominant manner [31]. Still, only very limited evidence exists on associations between SNPs other than the PNPLA3 and TM6SF2 variants and MASLD in lean individuals.

Notably, genome-wide association studies have highlighted numerous other well-validated or newly identified loci associated with liver injury and complications in MASLD, which would be worthwhile to explore in lean populations [36,37]. Among those variants were alcohol dehydrogenase 1B (ADH1B), apolipoprotein E (APOE), cordon-bleu WH2 repeat protein-like 1 (COBLL1)/growth factor receptor-bound protein 14 (GRB14), insulin receptor (INSR), glycerol-3-phosphate acyltransferase (GPAM), mitochondrial amidoxime-reducing component 1 (MARC1), microsomal triglyceride transfer protein large subunit (MTTP), patatin-like phospholipase domain-containing protein 2 (PNPLA2), receptor-type tyrosine-protein phosphatase δ (PTPRD), sterol regulatory element-binding transcription factor 1 (SREBF1), transmembrane channel like 4 (TMC4)/membrane-bound O-acyltransferase domain containing 7 (MBOAT7), torsin family 1 member B (TOR1B), and tribbles homolog 1 (TRIB1) [36,37]. The findings concerning those variants are promising for the stratification of lean patients with MASLD, particularly in disease management. As each patient tends to be influenced by a different polygenic set, the concept of using a polygenic risk score to stratify disease risk is reasonable [38]. A recent study, for instance, identified a strong association between a high polygenic risk score, calculated from the predisposing variants of PNPLA3, TM6SF2, GCKR, and FTO, and MASLD-related liver injury in overweight/high BMI patients [39]. While combining multiple genetic variants associated with MASLD or liver injury may improve the identification of MASLD risk compared to only assessing single variants, whether polygenic risk contributes more to MASLD risk in lean individuals than in overweight/high BMI individuals is still unknown.

Altogether, MASLD in lean individuals is hypothetically ascribed to the polygenic influence of the disease-associated SNPs; focusing on each variant by disregarding the others as a whole is insufficient to fill in gaps in the genetic basis of the disease. As preliminary as the findings are, the exact mechanisms of those SNPs in the pathogenesis of MASLD in lean individuals remain to be confirmed.

### 4.2. Metabolic Risk Profiles in Lean Individuals with MASLD

Lean individuals with MASLD have a “more favorable” metabolic risk profile than their non-lean counterparts. Despite having a BMI below the overweight cut-off, lean individuals with MASLD had a higher BMI and WC than lean individuals without MASLD [24,25,26,27,28,29,30,31,32,40,41,42,43,44,45,46,47,48,49,50,51,52]. Further, visceral adiposity is higher in lean individuals with MASLD than in lean individuals without MASLD, though visceral adiposity is still lower in lean MASLD than in high-BMI MASLD [12,29,40,43]. There is no consistent difference in total cholesterol (TC) and LDL between high-BMI MASLD, lean MASLD, and lean individuals without MASLD. In contrast, both TG and HDL are the highest and lowest in high-BMI patients with MASLD, respectively. The difference in TC and LDL levels between all groups was still inconclusive [24,25,26,27,28,29,30,31,32,40,41,42,43,44,45,46,47,48,49,50,51,52]. In contrast, TG and HDL levels increased stepwise from lean individuals without MASLD to those with MASLD and overweight/obese individuals without MASLD to those with MASLD [24,25,26,27,28,29,30,31,32,40,41,42,43,44,45,46,47,48,49,50,51,52]. Collectively, visceral obesity, as evaluated by increased WC, could contribute to the pathogenesis of MASLD in lean individuals. Also, impaired lipid metabolism can be evaluated in these patients by an increase in TG and a decrease in HDL.

Dysregulated glucose metabolism or insulin resistance—designated by an increased level of either fasting blood sugar (FBS), hemoglobin A1c (HbA1c), or resting insulin—is also a critical factor involved in metabolic dysregulation [53,54]. Of note, impaired FBS (prediabetes) and homeostasis model assessment of insulin resistance (HOMA-IR) were highest in non-lean individuals with MASLD, followed by lean individuals with MASLD and lean individuals without MASLD, respectively [24,26,27,28,29,31,32,40,41,42,43,44,46,47,48,49,50,52]. Nevertheless, the resting insulin and HbA1c levels varied and were inconclusive between lean and non-lean individuals with or without MASLD [24,25,27,30,42,43,46,47,49,50,51]. Thus, further addressing whether there are changes in levels of resting insulin and HbA1c in lean individuals with MASLD compared to the other groups, especially lean individuals without MASLD, is required. All of the above supports the idea that insulin resistance plays a critical role in developing MASLD in lean individuals.

DM, HTN, HLD, and metabolic syndrome are deliberated as explicit signs of metabolic dysregulation [55]. It is unsurprising that the number of individuals afflicted by each disease was highest in non-lean individuals with MASLD, followed by lean individuals with MASLD and lean individuals without MASLD [24,25,26,27,28,30,31,32,40,43,44,45,47,48,51,52,56,57]. Notably, according to the updated guidelines on the diagnosis and management of MASLD in lean individuals, those diagnosed with DM should then be investigated for MASLD [58,59]. Additionally, uric level—a CV risk-related parameter—showed similar trends. In contrast, the status of chronic kidney disease and its corresponding creatinine level were not investigated in previous studies [25,29,30]. Generally, the overall features of metabolic profiles in lean individuals with MASLD range between those found in lean individuals without MASLD and non-lean patients with MASLD.

### 4.3. Cardiovascular Risk Factors in Lean Individuals with MASLD

Not only are SNPs able to dysregulate lipid metabolism, resulting in MASLD in lean individuals, but an unhealthy lifestyle focusing on chronic energy imbalance—excess dietary intake over energy expenditure—is a major component in the pathogenesis of the disease. In this case [29], CV risk and its corresponding parameters have been accepted as criteria for diagnosing metabolic dysfunction [60,61,62]. Contrary to a non-lean individual who is at high CV risk, Table 2 lists CV risk factors and metabolic profiles found in lean patients with MASLD. Among CV risk factors, representative parameters for MASLD in a lean individual comprise WC, TC, TG, HDL, FBS, and blood pressure. All of which are convenient and available in a clinical setting. Suppose a lean individual presents with at least one increase in either WC, TC, TG, or FBS level, a decrease in HDL level, and an underlying DM, HTN, or HLD disease. In that case, this patient should be suspected of steatotic liver disease and require further investigation to diagnose MASLD, particularly in those older than 40 years old with type-2 DM [59].

### 4.4. Lifestyles, Smoking, Physical Activity, and Sarcopenia

MASLD in lean individuals culminates in an unhealthy lifestyle. Regarding daily energy expenditure, lean individuals with MASLD were inclined to have a lower frequency of exercise than those without MASLD and even non-lean individuals without MASLD. [41,44,45,47,49] However, the frequency of exercise varied between lean and non-lean patients with MASLD [41,44,45,47,49]. Additionally, skeletal muscle mass was highest in lean individuals without MASLD, followed by lean and non-lean individuals with MASLD, respectively [42,43]. It is well established that sarcopenia—a reduction in skeletal muscle mass—usually results from low physical activity, inferring that sarcopenia could be one of the features underlying metabolic dysfunction in MASLD [63,64]. It is not surprising that the thin outside but fat inside (TOFI) phenotype—high waist-to-hip ratio with a high level of truncal subcutaneous fat deposition but still within a normal BMI—represents the body composition of MASLD in lean individuals, as exemplified by the Indian population [65]. On one hand, anti-inflammatory adiponectin and inflammatory-inducing leptin, both of which can also be considered in terms of the adiponectin-to-leptin ratio, were examined to determine the status of adipose tissue inflammation or lipotoxicity [66,67,68]. Accumulated evidence addressing these adipocyte cytokines also substantiated that lean individuals with MASLD had a more severe dysregulated lipid metabolism than lean individuals without MASLD [24,26,27,49]. In addition, in sequence, levels of systemic inflammatory cytokines (e.g., IL-6 and TNF-alpha) and their associated markers (e.g., C-reactive protein and ferritin) in circulation tend to be highest in non-lean individuals with MASLD, followed by lean individuals with MASLD and lean individuals without MASLD, respectively [24,27,28,29,41,44,48,49,50]. Next, current smoking, another modifiable, might not be necessary for driving the pathogenesis of the disease due to inconclusive findings between all groups [26,30,41,43,44,45,47,48,49,50]. Still, there is a lack of research investigating the actual effect of smoking on the disease, and, in addition to current smoking, the history of previous smoking should have been explored as well. An unhealthy lifestyle, particularly low physical activity, is a major contributor to MASLD in lean individuals.

### 4.5. Gut Microbiome

Aside from the aspect of energy expenditure, excess dietary intake of high-fat and sugar components accompanied with dysbiosis of the gut microbiota has long been accounted for in the pathogenesis of MASLD [18]. Yet, evidence comprehensively assessing nutrition and the transformation of gut microbiota in lean patients with MASLD is still scanty. One study demonstrated that either a cholesterol-rich or high-sucrose diet was incapable of inducing MASLD in a murine model [23]. Notably, in this study, a cholesterol-rich diet preserved weight, while the latter resulted in weight gain, indicating that the nutritional composition may play a role in determining weight changes in the case of MASLD [23]. Moreover, the gut microbiota of lean mice with MASLD had a higher abundance of Bacteroidetes and a lower amount of Firmicutes compared to that of non-lean mice with MASLD [23]. Collectively, this study highlighted causal relationships between specific unhealthy diets and the development of distinct profiles of gut dysbiosis, as well as the resulting weight phenotype in MASLD. Furthermore, recent studies confirmed that the microbial compositions of gut dysbiosis were distinct between lean and non-lean individuals with MASLD [69,70]. In a clinical study, colonoscopy fecal microbiota transplantation, in which feces were derived from a healthy subject, improved gut dysbiosis and attenuated the steatotic liver of lean individuals with MASLD better than those of non-lean individuals with MASLD [70]. It implies that lean individuals’ gut dysbiosis may be mechanistically associated with MASLD. Therefore, gut microbial manipulation to reconstruct symbiotic microbiota seems to provide benefits for mitigating MASLD in lean individuals.

**Figure 1:**
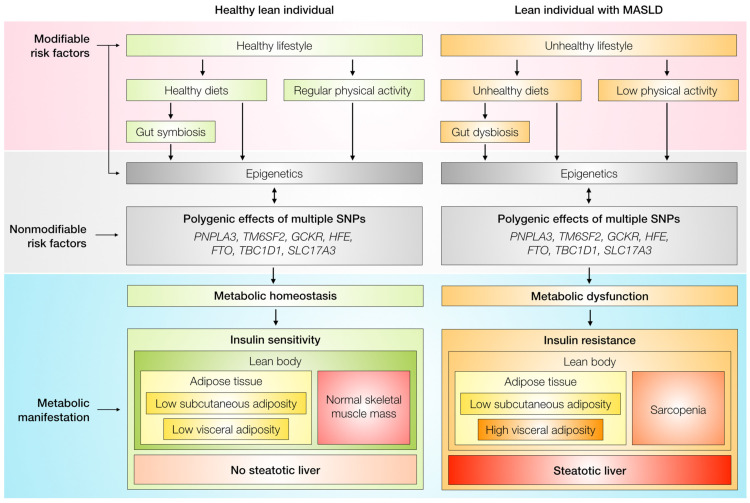
Schematic pathogenesis of MASLD in a lean individual. FTO, fat mass and obesity-associated alpha-ketoglutarate dependent dioxygenase; GCKR, glucokinase regulatory protein; HFE, human homeostatic iron regulator; MASLD, metabolic dysfunction-associated steatotic liver disease; PNPLA3, patatin-like phospholipase domain-containing 3; SLC17A3, solute carrier family 17 member 3; SNPs, single-nucleotide polymorphisms; TBC1D1, TBC1 domain family member 1; and TM6SF2, transmembrane 6 superfamily member 2.

MASLD in a lean individual is intertwined by nonmodifiable polygenic factors with modifiable epigenetic and lifestyle-related factors, of which the latter is exemplified by, but not limited to, unhealthy intake, gut dysbiosis, and low physical activity. Then, these factors concurrently promote the triad of metabolic dysregulation—insulin resistance, high visceral adiposity, and sarcopenia—eventually instigating steatotic liver disease with the TOFI body composition. Additionally, diabetic status is also considered an important predictor of outcomes in lean individuals with MASLD [71]. Still, the mechanisms underlying whether patients with these factors will develop the TOFI phenotype are poorly understood. One of the widely accepted theories is metabolic adaptation [18]. In contrast to their non-lean counterparts, lean individuals may have compensatory adaptations against the development of obesity even under the influence of those obesogenic factors [18]. These mechanisms, for example, were attributed to increases in levels of bile acid, fibroblast growth factor 19, and Farnesoid X receptor activity, as well as distinct profiles of the gut microbiota [23]. Given that lysine plays an important role in mediating visceral fat accumulation, lean individuals with MASLD had a higher level of this amino acid than their non-lean counterparts, possibly explaining why the lean patients resist the development of obesity. Differences in metabolic adaptation may partially explain the pathophysiology and provide options for therapy [24]. Figure 1 schematically overviews the pathogenesis of MASLD in lean individuals as compared to a healthy lean individual.

## 5. Natural History of MASLD in Lean Individuals

Initially, lean individuals with MASLD have better metabolic profiles, CV risks, liver function tests, and histopathology than non-lean patients. Table 3 summarizes the characteristics of liver function tests, histopathology, and liver-related complications of MASLD in lean individuals compared to non-lean individuals. However, in the end, the clinical course of lean patients progresses overtly, and, eventually, they experience a similar fate or even more severe outcomes than their non-lean counterparts during follow-up. These include liver-related complications, CV diseases (CVD), non-liver cancers, and overall mortality.

Lean individuals with MASLD at diagnosis have a lower baseline fibrosis stage and lower transaminase levels compared to their non-lean counterparts [24,25,26,27,28,29,30,41,42,44,45,46,47,48,49,51]. In concordance with the liver enzymes, histopathological studies found that lean individuals with MASLD had less severe lobular inflammation, steatosis, ballooning, and fibrosis than their counterparts [25,26,27,28,46,48]. However, during follow-up, the lean individuals also develop liver-related complications—cirrhosis, HCC, liver decompensation, and death—similar to the non-lean individuals [28,30]. In contrast, a French cohort study reported that having a lean status increased the risk of advanced liver fibrosis, cirrhosis, and the incidence of hepatic events [45]. Examining liver function tests and histopathology provided a clinical picture of MASLD prior to the progression of the disease.

Besides liver-related complications, lean individuals are equally or somewhat more affected by CVD, non-liver cancers, and overall mortality compared to non-lean individuals. The incidence of CVD remains similar across patients with MASLD, regardless of BMI status [28,30]. In contrast, in the French cohort, lean individuals with MASLD had an upward trend in CVD incidence compared with non-lean patients. However, this is applicable only to patients with cirrhosis and advanced fibrosis and not to those without these conditions [45]. Of note, a French study defined MASLD based on fatty liver index (FLI) criteria, which could partially explain the difference in incidents of liver cirrhosis, liver decompensation, and CVD from other studies [45]. Regarding liver cancer, patients with MASLD also have a heightened risk of developing cancer [62]. In a similar fashion to CVD, there was an indifferent incidence of non-liver cancers between both groups [28,30]. Interestingly, one study found that the incidence of chronic kidney disease increased in lean individuals with MASLD compared to their non-lean counterparts [45]. Notably, accumulated evidence has shown that overall mortality, of which the other causes apart from liver-related complications included CV events and any other cancers, was higher in lean patients than non-lean patients [30,45]. In contrast, one study showed that overall mortality was indifferent between both groups at the end [28].

Research on mortality in lean individuals with MASLD has yielded contradictory results, likely due to variations in study populations and methodologies [28,30,57]. It is important to note that lean individuals may have underlying comorbidities that contribute to their mortality, rather than MASLD itself.

Still, mechanistic evidence underlying how MASLD overtly progresses in lean individuals and, finally, undergoes a similar fate to those non-lean individuals during follow-up has been poorly understood. Some studies postulated that, contrary to non-lean individuals, MASLD in lean individuals might be a paradoxical outcome of metabolic maladaptation regarding weight control systems, adiposity distribution, core energy metabolism, and impaired inflammatory and fibrogenic responses [18].

**Figure 2:**
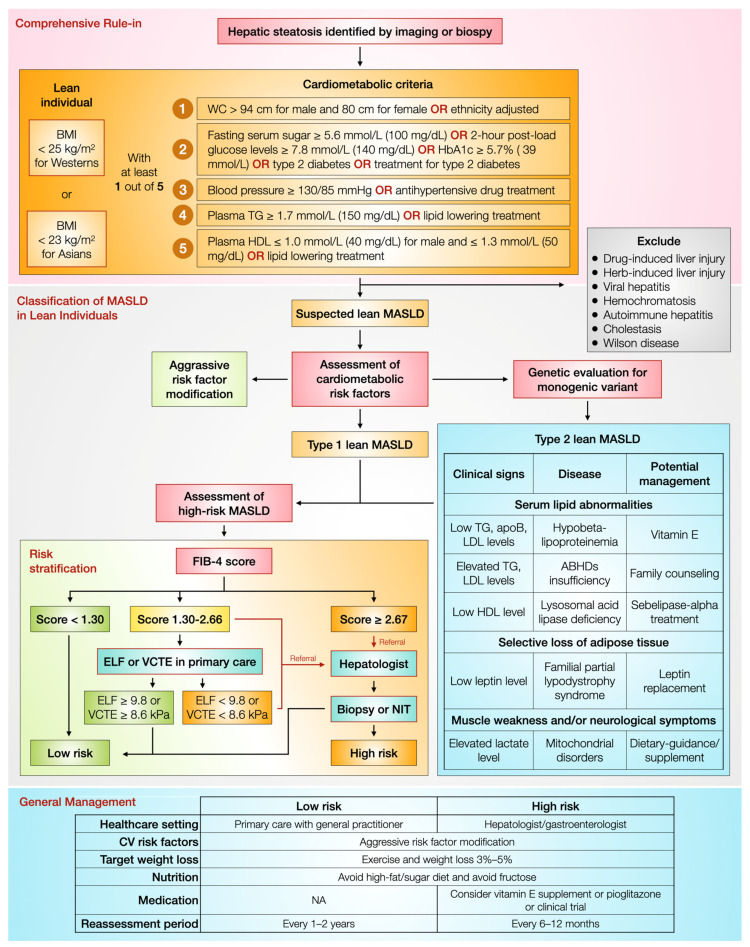
The proposed current approach and management for MASLD in a lean individual. ABHDs, alpha/beta hydrolase domain-containing proteins; apoB, apolipoprotein B; BMI, body mass index; CV, cardiovascular; ELF, enhanced liver fibrosis test; FIB, fibrosis; HbA1c, hemoglobin A1c; HDL, high-density lipoprotein; LDL, low-density lipoprotein; MASLD, metabolic dysfunction-associated steatotic liver disease; NA, not available; NIT, non-invasive tests; TG, triglyceride; WC, weight circumference; and VCTE, vibration–controlled transient elastography. (Adapted from [59,72]).

As lean patients with MASLD tend to face a similar clinical course or ever more severe liver and non-liver complications and overall mortality than their non-lean counterparts, this section collectively summarizes the current expert consensus on how to clinically approach and manage MASLD in lean individuals [59,72,73]. Figure 2 proposes the current approach and management for lean patients with MASLD.

The first step is to identify lean patients with MASLD based on the current diagnostic criteria: individuals with BMI less than 25 and 23 for the Western and Asian populations, respectively, with evidence of hepatic steatosis on imaging or liver biopsy, with any of these cardiometabolic criteria, an abnormal metabolic profile including WC, TG, fasting blood sugar, blood pressure, and HDL, or metabolic diseases (HLP, HTN, and DM), or any medication for these diseases, are qualified for MASLD in lean individuals diagnosis [59]. Next, other liver disease etiologies, whose pathology might overlap with steatotic liver diseases, must be excluded. Those include drug/herb-induced liver injury, viral hepatitis, hemochromatosis, autoimmune hepatitis, and Wilson disease. Furthermore, the extent of alcohol intake is frequently a complicating factor for patients with MASLD, often due to inaccuracies in reporting alcohol consumption levels [19]. There is increasing evidence highlighting a growing trend in both alcohol use and alcohol-associated liver disease [74,75]. Therefore, identifying patients with lean MASLD could possibly be enhanced by excluding those with alcohol-associated liver disease, potentially through the detection of alcohol biomarkers [76,77].

MASLD in lean individuals is classified into two types [72]. Type 1 lean MASLD is more typical in terms of having visceral adiposity, increased WC, and a metabolic risk profile. In addition, they have common genetic variants, such as *PNPLA3* and *TM6SF2* [72]. In contrast, type 2 lean MASLD does not have visceral adiposity or a typical metabolic risk profile. Notably, genetic evaluation to unveil a monogenic disorder is necessitated for type 2 of MASLD, which requires disease-specific treatment [72].

Regardless of the types of MASLD, the fibrosis-4 (FIB-4) score is calculated to initially stratify the risk of liver fibrosis: low score (<1.30), intermediate score (1.30–2.66), and high score (≥2.67) [59]. Dissimilar to the low score indicating a low risk of having liver fibrosis, the intermediate-to-high score necessitates the confirmation of liver fibrosis via liver biopsy or other non-invasive tests by a specialist. In the case of the intermediate score, if the enhanced liver fibrosis test (ELF) or the vibrant-controlled transient elastography (VCTE) is available in a primary care setting, the patients should be further evaluated using one of the two tests. ELF ≥ 9.8 and VCTE ≥ 8.6 indicate a low risk of liver fibrosis, whereas scores below these values mandate referral to a specialist.

In general, for both high-risk and low-risk patients, the key to aggressive metabolic risk factor modification is a lifestyle modification that balances dietary intake and daily expenditure. Of note, high-fat/sugar and high-fructose diets should be avoided, while the goal of increasing exercise is to reduce body weight by at least 3–5% [59,73]. In terms of medications, there is no Food and Drug Administration-approved medication for lean individuals with MASLD. However, vitamin E may be considered in lean individuals with biopsy-confirmed MASLD without DM or cirrhosis, and oral pioglitazone 30 mg daily may be prescribed in lean patients with biopsy-confirmed MASLD without cirrhosis [59]. Due to MASLD being able to overtly progress, a patient should be reassessed every 1–2 years for low risk and 6–12 months for high risk [59]. Per new AASLD MASLD guidance in 2023, the management of lean individuals with MASLD emphasizes dietary modifications and exercise to improve insulin resistance since recommending weight loss may not be appropriate for some lean patients with MASLD [78].

Besides the management of MASLD itself, it needs to be mentioned that MASLD increases the risk of liver and other gastrointestinal cancers [79]. Therefore, preventive measures need to be considered. Weight loss, either through lifestyle modification or diet control, decreases the risk of MASLD and hepatocellular carcinoma [80,81].

## 6. Conclusions

Our review revealed that MASLD affects a significant proportion of lean individuals, and these patients experience significant complications, both related to the liver and non-liver issues. Consequently, screening for MASLD should not solely rely on obesity as a criterion; instead, the focus should be on assessing the metabolic health of patients, such as those with DM. Furthermore, genetic testing such as whole genome sequencing may have a role in uncovering underlying monogenic disorders in lean individuals with MASLD, particularly in those without metabolic risk profiles.

## Figures and Tables

**Table 1:** MASLD-associated genes and their single-nucleotide polymorphism in lean individuals.

Associated Gene in Lean MASLD	SNP ID	Function	Alleles	Genotype	Country	Findings	Ref.
*PNPLA3*	rs738409	Missense variant (C>G, I148M)	C/G	CC, CG, GG	Australia	LM and NLM had indifferent GG frequency	[23]
					Austria	CC/(CG + GG) ratio decreased in LNM but was equal to NLM compared to LM	[24]
					Hong Kong	LM and NLM had indifferent CC or CG + GG frequency	[25]
					Italy	LM and NLM had an indifferent GG frequency	[26]
					Japan	LM and NLM had an indifferent G allele frequency	[27]
					Italy, UK, Spain, Australia	LM and NLM had indifferent CC, CG, and GG frequencies	[28]
					UK	G allele frequency increased in LM compared with LNM G allele frequency increased in NLM compared with NLNM	[29]
					USA	GG frequency was not only associated with cirrhosis in LM and NLM but other liver-related complications in NLM	[30]
*TM6SF2*	rs58542926	Missense variant (C>T, E167K)	C/T	CC, CT, TT	Australia	T allele frequency increased in LM compared with LNM	[23]
					Austria	LM, LNM, and NLM had an indifferent CC/(CT + TT) ratio	[24]
					Hong Kong	LM and NLM had indifferent CC or CT + TT frequencies	[25]
					Italy	CC + TT increased in LM compared with NLM	[26]
					UK	T allele frequency increased in LM compared with LNM T allele frequency increased in NLM compared with NLNM	[29]
*NCAN*	rs2228602	Missense Variant (C>T, D704=)	C/T	CC, CT, TT	Austria	LM, LNM, and NLM had an indifferent CC/(CT + TT) ratio	[24]
*GCKR*	rs6834314	NR	C/T	CC, CT, TT	Austria	LM, LNM, and NLM had an indifferent CC/(CT + TT) ratio	[24]
	rs1260326	Missense variant (T>C, P466L)	T/C	TT, TC, CC	China	Increased C allele frequency was associated with increased waist circumference	[31]
	rs780093	Intron variant	T/C	TT, TC, CC	China	C allele frequency decreased in LM compared with LNM	[31]
	rs780094	Intron variant	T/C	TT, TC, CC	China	C allele frequency decreased in LM compared with LNM	[31]
*LYPLAL1*	rs12137855	Intron Variant	C/T	CC, CT, TT	Austria	LM, LNM, and NLM had an indifferent CC/(CT + TT) ratio	[24]
*TBC1D1*	rs2279028	Upstream gene variant	A/G	AA, AG, GG	China	An allele frequency increased in LM compared with LNM Increased AA or GG frequency was associated with low HDL	[32]
*HFE*	rs1800562	Missense variant (G>A, C282Y)	G/A	GG, GA, AA	UK	An allele frequency increased in LM compared with LNM	[29]
*SLC17A3*	rs9348697	Upstream gene variant	C/T	CC, CT, TT	UK	T allele frequency increased in LM compared with LNM	[29]
*FTO*	rs1421085	Intron variant	T/C	TT, TC, CC	China	Increased CC frequency was associated with high LDL in LM	[33]
	rs3751812	Intron variant	G/T	GG, GT, TT	China	Increased TT frequency was associated with high LDL in LM	[33]
	rs8050136	Intron variant	C/A	CC, CA, AA	China	Increased AA frequency was associated with high LDL in LM	[33]
	rs9939609	Intron variant	T/A	TT, TA, AA	China	Increased AA frequency was associated with high LDL in LM	[33]

Abbreviations: FTO, fat mass and obesity-associated alpha-ketoglutarate-dependent dioxygenase; GCKR, glucokinase regulatory protein; HFE, human homeostatic iron regulator; LYPLAL1, lysophospholipase-like 1; LNM, healthy lean individual without metabolic dysfunction-associated steatotic liver disease; LM, lean individual with metabolic dysfunction-associated steatotic liver disease; MASLD, metabolic dysfunction-associated steatotic liver disease; NCAN, neurocan; NLM, non-lean individual with metabolic dysfunction-associated steatotic liver disease; NLNM, non-lean individual with non-metabolic dysfunction-associated steatotic liver disease; PNPLA3, patatin-like phospholipase domain-containing 3; rs, reference single-nucleotide polymorphism; SLC17A3, solute carrier family 17 member 3; SNP ID, single-nucleotide polymorphism identification number; TBC1D1, TBC1 domain family member 1; and TM6SF2, transmembrane 6 superfamily member 2.

**Table 2:** Cardiometabolic risk profiles of a lean individual with MASLD.

Year	Country	LNM *n* (%M)	NLNM *n* (%M)	LM *n* (%M)	NLM *n* (%M)	Age of LM (Year) *	Diagnostic Method of SLD	Cardiometabolic Risk								Ref.
								WC	FBS	HbA1c	TG	HDL	DM	HTN	HLD	
2021	Austria	254 (32.7)	NR	169 (52.7)	471 (69.2)	59.6 ± 8.6	U/S and TE	↑ ^a^, ↓ ^c^	NR	NR	↑ ^a^, ↓ ^c^	↓ ^a^, ↑ ^c^	↑ ^a^, ↓ ^c^	↑ ^a^, ↓ ^c^	↑ ^a^, ↓ ^c^	[40]
2021	Austria	892 (34.2)	NR	205 (56.6)	1141 (64.6)	60.3 ± 10.2	U/S	↑ ^a^, ↓ ^c^	NR	NR	↑ ^a^, ↓ ^c^	↓ ^a^, ↑ ^c^	↑ ^a^, ↓ ^c^	↑ ^a^, ↓ ^c^	↑ ^a^, ↓ ^c^	[40]
2017	Austria	71 (54.3)	NR	55 (47.3)	61 (47.5)	61 (12.5)	U/S	= ^a^, ↓ ^c^	↑ ^a^, ↓ ^c^	= ^a^, ↓ ^c^	↑ ^a^, ↓ ^c^	↓ ^a^, ↑ ^c^	↑ ^a^, ↓ ^c^	NR	NR	[24]
2020	Brazil	3372 (54.8)	2880 (82.2)	349 (88)	2536 (92.1)	44 ± 9	U/S	↑ ^a^, ↓ ^b, c^	↑ ^a,b^, ↓ ^c^	NR	↑ ^a,b^, ↓ ^c^	↓ ^a^, = ^b^, ↑ ^c^	NR	NR	NR	[41]
2023	China	NR	NR	262 (24.4)	1043 (35.3)	NR	U/S	↓ ^c^	↓ ^c^	↓ ^c^	↓ ^c^	↑ ^c^	NR	NR	NR	[42]
2023 2022	China	216 (41.2)	NR	106 (35.8)	NR	72.54 ± 6.05	U/S	↑ ^a^	↑ ^a^	NR	↑ ^a^	↓ ^a^	= ^a^	↑ ^a^	= ^a^	[31,32]
2022	China	743 (66.8)	NR	369 (33.2)	NR	53.45 ± 10.91	U/S	↑ ^a^	= ^a^	= ^a^	↑ ^a^	↓ ^a^	↓ ^a^	NR	NR	[43]
2022	China	19,605 (69.3)	31,105 (77.7)	1543 (75)	21,654 (78)	53.6 ± 11.4	U/S	↑ ^a^, ↓ ^b, c^	↑ ^a,b^, ↓ ^c^	NR	↑ ^a,b^, ↓ ^c^	= ^a^, ↑ ^b, c^	↑ ^a,b^, ↓ ^c^	↑ ^a,b^, ↓ ^c^	NR	[44]
2023	France	NR	NR	3664 (43.7)	22,089 (69.6)	45.1 (NR)	U/S	↓ ^c^	NR	NR	↓ ^c^	NR	↓ ^c^	NR	↓ ^c^	[45]
2017	Hong Kong	NR	NR	72 (45.8)	235 (58.7)	54 ± 11	H and TE	↓ ^c^	= ^c^	= ^c^	= ^c^	= ^c^	= ^c^	↓ ^c^	NR	[25]
2022	India	NR	NR	267 (NR)	1006 (NR)	43 (19)	H	NR	↓ ^c^	= ^c^	= ^c^	= ^c^	NR	NR	NR	[46]
2017	Italy	NR	NR	143 (72)	526 (72)	46 ± 13	H	↓ ^c^	NR	NR	= ^c^	↑ ^c^	↓ ^c^	↓ ^c^	NR	[26]
2023	Japan	NR	NR	86 (50.0)	695 (53.0)	57.5 (62)	H	NR	NR	↓ ^c^	↓ ^c^	NR	= ^c^	↓ ^c^	↓ ^c^	[27]
2021	Korea	2987 (30.9)	NR	525 (57.7)	1274 (40.3)	60.5 ± 10.8	Predictive model	↑ ^a^, ↓ ^c^	↑ ^a,c^	NR	↑ ^a,c^	↓ ^a,c^	↑ ^a,c^	↑ ^a^, = ^c^	NR	[47]
2018	Sweden	NR	NR	123 (57.7)	523 (63.3)	51.4 ± 13.4	H	NR	↓ ^c^	NR	↓ ^c^	NR	↓ ^c^	= ^c^	= ^c^	[48]
2023	Taiwan	217 (20.7)	NR	105 (34.3)	200 (56.5)	42.96 ± 11.59	U/S	↑ ^a^, ↓ ^c^	= ^a^, ↓ ^c^	NR	↑ ^a^, ↓ ^c^	↓ ^a^, ↑ ^c^	NR	NR	NR	[49]
2023	UK	10,266 (38.6)	2245 (45.6)	631 (58.0)	2115 (57.35)	67.74	MRI	↑ ^a^, ↓ ^b, c^	↑ ^a,b^, = ^c^	NR	↑ ^a^, ↓ ^b,c^	↓ ^a^, ↑ ^b,c^	NR	NR	NR	[29]
2022	UK	NR	NR	136 (53)	871 (60)	55.8 ± 7.4	NR	↓ ^c^	= ^c^	↓ ^c^	↑ ^c^	↑ ^c^	NR	NR	NR	[50]
2023	USA	NR	NR	430 (34.9)	2980 (44.0)	NR	Imaging, H, and TE	NR	NR	NR	NR	NR	↓ ^c^	= ^c^	= ^c^	[56]
2023	USA	NR	NR	2137 (41.2)	16,457 (47.9)	51.0 (27)	Imaging and H	NR	NR	↓ ^c^	↓ ^c^	↑ ^c^	↓ ^c^	↓ ^c^	↓ ^c^	[30]
2022	USA	NR	NR	414 (34.1)	4420 (47.0)	51.5 ± 18.0	Imaging, H, and TE	NR	NR	NR	NR	NR	↓ ^c^	↓ ^c^	↓ ^c^	[57]
2021	USA	NR	NR	433 (41.1)	2953 (41.4)	58.5 ± 13.1	H	NR	NR	= ^c^	↓ ^c^	↑ ^c^	↓ ^c^	↓ ^c^	↓ ^c^	[51]
2014	USA	NR	NR	125 (NR)	965 (NR)	NR	Imaging and H	↓ ^c^	NR	NR	↓ ^c^	↑ ^c^	↓ ^c^	↓ ^c^	↓ ^c^	[52]
2021	Italy, UK, Spain, Australia	NR	NR	195 (75.4)	1144 (37.3)	45 (19)	U/S and TE	↓ ^c^	↓ ^c^	NR	↓ ^c^	= ^c^	↓ ^c^	NR	NR	[28]

* Age expressed as mean ± standard deviation or median (interquartile range); ↑ increase; ↓ decrease; = not remarkable difference; ^a^ compared to LNM in its study; ^b^ compared to NLNM in its study; and ^c^ compared to NLM (high BMI individuals) in its study. Abbreviations: DM, diabetes mellitus; FBS, fasting blood sugar; H, histology; HbA1c, hemoglobin A1c; HDL, high-density lipoprotein cholesterol; HLD, hyperlipidemia; HTN, hypertension; LNM, lean individual with non-metabolic dysfunction-associated steatotic liver disease; LM, lean individual with metabolic dysfunction-associated steatotic liver disease; M, male; MASLD, metabolic dysfunction-associated steatotic liver disease; MRI, magnetic resonance imaging; NLM, non-lean individual with metabolic dysfunction-associated steatotic liver disease; NLNM, non-lean individual without metabolic dysfunction-associated steatotic liver disease; NR, not report; SLD, steatotic liver disease; TE, transient elastography; TG, total triglyceride; U/S, ultrasound; and WC, waist circumference.

**Table 3:** Liver function, histopathology, and liver-related complications of MASLD in lean individuals compared with non-lean individuals.

Year	Study Design	Country	LM *n* (%M)	NLM *n* (%M)	Age of LM (Year) *	Diagnostic Method of SLD	Liver Function (LM vs. NLM)				Histopathological Severity of SLD (LM vs. NLM)	Liver-Related Complications (LM vs. NLM)	Ref.
							Hepatocyte Integrity	Biliary Excretory Function	Synthetic Function	PLT			
2017	C	Austria	55 (47.3)	61 (47.5)	61 (12.5)	U/S	=	=	NR	NR	NR	NR	[24]
2020	P	Brazil	349 (88)	2536 (92.1)	44 ± 9	U/S	↑	↑	NR	NR	NR	NR	[41]
2023	P	China	262 (24.4)	1043 (35.3)	NR	U/S	=	NR	NR	NR	NR	=	[42]
2022	P	China	1543 (75)	21,654 (78)	53.6 ± 11.4	U/S	↑	NR	NR	NR	NR	↑	[44]
2023	P	France	3664 (43.7)	22,089 (69.6)	45.1 (NR)	U/S	↓	↓	NR	NR	NR	↑	[45]
2017	P	Hong Kong	72 (45.8)	235 (58.7)	54 ± 11	H and TE	=	↓	NR	NR	=	↓	[25]
2022	C	India	267 (NR)	1006 (NR)	43 (19)	H	↓	=	NR	NR	=	NR	[46]
2017	C	Italy	143 (72)	526 (72)	46 ± 13	H	=	NR	=	↑	↓	NR	[26]
2023	R	Japan	86 (50.0)	695 (53.0)	57.5 (62)	H	↑	NR	=	=	↓	=	[27]
2021	C	Korea	525 (57.7)	1274 (40.3)	60.5 ± 10.8	Predictive model	↓	↓	NR	=	NR	NR	[47]
2018	R	Sweden	123 (57.7)	523 (63.3)	51.4 ± 13.4	H	↑	=	↑	=	↓	↑	[48]
2023	C	Taiwan	105 (34.3)	200 (56.5)	42.96 ± 11.59	U/S	↑	NR	NR	NR	NR	NR	[49]
2023	R	UK	631 (58.0)	2115 (57.35)	67.74	MRI	↑	↑	NR	NR	NR	NR	[29]
2023	R	USA	430 (34.9)	2980 (44.0)	NR	NR	NR	NR	NR	NR	NR	=	[56]
2023	P	USA	2137 (41.2)	16,457 (47.9)	51.0 (27)	Imaging, H, and TE	↑	NR	NR	↓	↓	=	[30]
2022	R	USA	414 (34.1)	4420 (47.0)	51.5 ± 18.0	Imaging and H	NR	NR	NR	NR	NR	=	[57]
2021	P	USA	433 (41.1)	2953 (41.4)	58.5 ± 13.1	Imaging, H, and TE	↑	=	↑	=	NR	↓	[51]
2021	P	Italy, UK, Spain, Australia	195 (75.4)	1144 (37.3)	45 (19)	Imaging and H	=	↓	=	=	↓	=	[28]

* Age expressed as mean ± standard deviation or median (interquartile range); ↑ increase; ↓ decrease; = not remarkable difference. Abbreviations: C, cross-sectional study; H, histology; HCC, hepatocellular carcinoma; LNM, lean individual with non-metabolic dysfunction-associated steatotic liver disease; LM, lean metabolic dysfunction-associated steatotic liver disease; M, male; MASLD, metabolic dysfunction-associated steatotic liver disease; MRI, magnetic resonance imaging; NLM, non-lean individual with metabolic dysfunction-associated steatotic liver disease; NLNM, non-lean individual without metabolic dysfunction-associated steatotic liver disease; NR, not report; P, prospective cohort study; PLT, platelet; R, retrospective cohort study; SLD, steatotic liver disease; TE, transient elastography; U/S, ultrasound6. Current approach and management for lean individuals.

## Data Availability

No new data were created or analyzed in this study. Data sharing is not applicable to this article.

## References

[1] Majumdar A., Tsochatzis E.A. (2020). Changing trends of liver transplantation and mortality from non-alcoholic fatty liver disease. Metabolism.

[2] Younossi Z.M., Golabi P., Paik J.M., Henry A., Van Dongen C., Henry L. (2023). The global epidemiology of nonalcoholic fatty liver disease (NAFLD) and nonalcoholic steatohepatitis (NASH): A systematic review. Hepatology.

[3] Danpanichkul P., Ng C.H., Muthiah M.D., Duangsonk K., Yong J.N., Tan D.J.H., Lim W.H., Wong Z.Y., Syn N., Tsusumi T. (2023). The silent burden of non-alcoholic fatty liver disease in the elderly: A global burden of disease analysis. Aliment. Pharmacol. Ther..

[4] Younossi Z.M., Stepanova M., Ong J., Trimble G., AlQahtani S., Younossi I., Ahmed A., Racila A., Henry L. (2021). Nonalcoholic Steatohepatitis Is the Most Rapidly Increasing Indication for Liver Transplantation in the United States. Clin. Gastroenterol. Hepatol..

[5] Danpanichkul P., Kongarin S., Permpatdechakul S., Polpichai N., Duangsonk K., Manosroi W., Chaiyakunapruk N., Mousa O.Y., Kim D., Chen V.L. (2023). The Surreptitious Burden of Nonalcoholic Fatty Liver Disease in the Elderly in the Asia-Pacific Region: An Insight from the Global Burden of Disease Study 2019. J. Clin. Med..

[6] Solomon A., Negrea M.O., Cipaian C.R., Boicean A., Mihaila R., Rezi C., Cristinescu B.A., Berghea-Neamtu C.S., Popa M.L., Teodoru M. (2023). Interactions between Metabolic Syndrome, MASLD, and Arterial Stiffening: A Single-Center Cross-Sectional Study. Healthcare.

[7] Wang A.Y., Dhaliwal J., Mouzaki M. (2019). Lean non-alcoholic fatty liver disease. Clin. Nutr..

[8] De A., Bhagat N., Mehta M., Taneja S., Duseja A. (2023). Metabolic dysfunction-associated steatotic liver disease (MASLD) definition is better than MAFLD criteria for lean patients with NAFLD. J. Hepatol..

[9] Francque S., Wong V.W. (2022). NAFLD in lean individuals: Not a benign disease. Gut.

[10] Duseja A., De A., Wong V. (2023). Special Population: Lean Nonalcoholic Fatty Liver Disease. Clin. Liver Dis..

[11] Eslam M., Chen F., George J. (2020). NAFLD in Lean Asians. Clin. Liver Dis..

[12] Ross R., Neeland I.J., Yamashita S., Shai I., Seidell J., Magni P., Santos R.D., Arsenault B., Cuevas A., Hu F.B. (2020). Waist circumference as a vital sign in clinical practice: A Consensus Statement from the IAS and ICCR Working Group on Visceral Obesity. Nat. Rev. Endocrinol..

[13] Seto W.K., Yuen M.F. (2017). Nonalcoholic fatty liver disease in Asia: Emerging perspectives. J. Gastroenterol..

[14] Ye Q., Zou B., Yeo Y.H., Li J., Huang D.Q., Wu Y., Yang H., Liu C., Kam L.Y., Tan X.X.E. (2020). Global prevalence, incidence, and outcomes of non-obese or lean non-alcoholic fatty liver disease: A systematic review and meta-analysis. Lancet Gastroenterol. Hepatol..

[15] Young S., Tariq R., Provenza J., Satapathy S.K., Faisal K., Choudhry A., Friedman S.L., Singal A.K. (2020). Prevalence and Profile of Nonalcoholic Fatty Liver Disease in Lean Adults: Systematic Review and Meta-Analysis. Hepatol. Commun..

[16] Tang A., Ng C.H., Phang P.H., Chan K.E., Chin Y.H., Fu C.E., Zeng R.W., Xiao J., Tan D.J.H., Quek J. (2023). Comparative Burden of Metabolic Dysfunction in Lean NAFLD vs Non-lean NAFLD—A Systematic Review and Meta-analysis. Clin. Gastroenterol. Hepatol..

[17] Maier S., Wieland A., Cree-Green M., Nadeau K., Sullivan S., Lanaspa M.A., Johnson R.J., Jensen T. (2021). Lean NAFLD: An underrecognized and challenging disorder in medicine. Rev. Endocr. Metab. Disord..

[18] Eslam M., El-Serag H.B., Francque S., Sarin S.K., Wei L., Bugianesi E., George J. (2022). Metabolic (dysfunction)-associated fatty liver disease in individuals of normal weight. Nat. Rev. Gastroenterol. Hepatol..

[19] Idalsoaga F., Kulkarni A.V., Mousa O.Y., Arrese M., Arab J.P. (2020). Non-alcoholic Fatty Liver Disease and Alcohol-Related Liver Disease: Two Intertwined Entities. Front. Med..

[20] Zheng M., Huang D.Q., Konkwo C., Agrawal S., Khera A.V., Loomba R., Vilarinho S., Ajmera V. (2023). Genomic analysis of lean individuals with NAFLD identifies monogenic disorders in a prospective cohort study. JHEP Rep..

[21] Youssefian L., Vahidnezhad H., Saeidian A.H., Pajouhanfar S., Sotoudeh S., Mansouri P., Amirkashani D., Zeinali S., Levine M.A., Peris K. (2019). Inherited non-alcoholic fatty liver disease and dyslipidemia due to monoallelic ABHD5 mutations. J. Hepatol..

[22] Adant I., Declercq M., Bird M., Bauters M., Boeckx N., Devriendt K., Cassiman D., Witters P. (2020). Two cases of non-alcoholic fatty liver disease caused by biallelic ABHD5 mutations. J. Hepatol..

[23] Chen F., Esmaili S., Rogers G.B., Bugianesi E., Petta S., Marchesini G., Bayoumi A., Metwally M., Azardaryany M.K., Coulter S. (2020). Lean NAFLD: A Distinct Entity Shaped by Differential Metabolic Adaptation. Hepatology.

[24] Feldman A., Eder S.K., Felder T.K., Kedenko L., Paulweber B., Stadlmayr A., Huber-Schonauer U., Niederseer D., Stickel F., Auer S. (2017). Clinical and Metabolic Characterization of Lean Caucasian Subjects with Non-alcoholic Fatty Liver. Am. J. Gastroenterol..

[25] Leung J.C., Loong T.C., Wei J.L., Wong G.L., Chan A.W., Choi P.C., Shu S.S., Chim A.M., Chan H.L., Wong V.W. (2017). Histological severity and clinical outcomes of nonalcoholic fatty liver disease in nonobese patients. Hepatology.

[26] Fracanzani A.L., Petta S., Lombardi R., Pisano G., Russello M., Consonni D., Di Marco V., Camma C., Mensi L., Dongiovanni P. (2017). Liver and Cardiovascular Damage in Patients With Lean Nonalcoholic Fatty Liver Disease, and Association With Visceral Obesity. Clin. Gastroenterol. Hepatol..

[27] Kawanaka M., Nishino K., Kawada M., Ishii K., Tanikawa T., Katsumata R., Urata N., Nakamura J., Suehiro M., Haruma K. (2023). Lean nonalcoholic fatty liver disease: Age-dependent differences in pathology, prognosis, and liver-related events. Hepatol. Res..

[28] Younes R., Govaere O., Petta S., Miele L., Tiniakos D., Burt A., David E., Vecchio F.M., Maggioni M., Cabibi D. (2022). Caucasian lean subjects with non-alcoholic fatty liver disease share long-term prognosis of non-lean: Time for reappraisal of BMI-driven approach?. Gut.

[29] Sun Z., Pan X., Tian A., Surakka I., Wang T., Jiao X., He S., Song J., Tian X., Tong D. (2023). Genetic variants in HFE are associated with non-alcoholic fatty liver disease in lean individuals. JHEP Rep..

[30] Wijarnpreecha K., Li F., Lundin S.K., Suresh D., Song M.W., Tao C., Chen V.L., Lok A.S.F. (2023). Higher mortality among lean patients with non-alcoholic fatty liver disease despite fewer metabolic comorbidities. Aliment. Pharmacol. Ther..

[31] Wu N., Li J., Zhang J., Yuan F., Yu N., Zhang F., Li D., Wang J., Zhang L., Shi Y. (2023). Waist circumference mediates the association between rs1260326 in GCKR gene and the odds of lean NAFLD. Sci. Rep..

[32] Wu N., Zhai X., Yuan F., Li J., Li D., Wang J., Zhang L., Shi Y., Ji G., He G. (2022). Genetic variation in TBC1 domain family member 1 gene associates with the risk of lean NAFLD via high-density lipoprotein. Front. Genet..

[33] Li J., Wu N., Yang Y., Zhai X., Yuan F., Zhang F., Yu N., Li D., Wang R., Wang J. (2023). Unique genetic variants of lean nonalcoholic fatty liver disease: A retrospective cohort study. BMC Endocr. Disord..

[34] Gruner Nielsen D., Andersen K., Sogaard Nielsen A., Juhl C., Mellentin A. (2021). Consistency between self-reported alcohol consumption and biological markers among patients with alcohol use disorder—A systematic review. Neurosci. Biobehav. Rev..

[35] Riazi K., Swain M.G., Congly S.E., Kaplan G.G., Shaheen A.A. (2022). Race and Ethnicity in Non-Alcoholic Fatty Liver Disease (NAFLD): A Narrative Review. Nutrients.

[36] Chen V.L., Du X., Chen Y., Kuppa A., Handelman S.K., Vohnoutka R.B., Peyser P.A., Palmer N.D., Bielak L.F., Halligan B. (2021). Genome-wide association study of serum liver enzymes implicates diverse metabolic and liver pathology. Nat. Commun..

[37] Chen Y., Du X., Kuppa A., Feitosa M.F., Bielak L.F., O’Connell J.R., Musani S.K., Guo X., Kahali B., Chen V.L. (2023). Genome-wide association meta-analysis identifies 17 loci associated with nonalcoholic fatty liver disease. Nat. Genet..

[38] Ajmera V., Wang N., Xu H., Liu C.T., Long M.T. (2023). Longitudinal association between overweight years, polygenic risk and NAFLD, significant fibrosis and cirrhosis. Aliment. Pharmacol. Ther..

[39] De Vincentis A., Tavaglione F., Jamialahmadi O., Picardi A., Antonelli Incalzi R., Valenti L., Romeo S., Vespasiani-Gentilucci U. (2022). A Polygenic Risk Score to Refine Risk Stratification and Prediction for Severe Liver Disease by Clinical Fibrosis Scores. Clin. Gastroenterol. Hepatol..

[40] Semmler G., Wernly S., Bachmayer S., Wernly B., Schwenoha L., Huber-Schonauer U., Stickel F., Niederseer D., Aigner E., Datz C. (2021). Nonalcoholic Fatty Liver Disease in Lean Subjects: Associations With Metabolic Dysregulation and Cardiovascular Risk-A Single-Center Cross-Sectional Study. Clin. Transl. Gastroenterol..

[41] Aneni E.C., Bittencourt M.S., Teng C., Cainzos-Achirica M., Osondu C.U., Soliman A., Al-Mallah M., Buddoff M., Parise E.R., Santos R.D. (2020). The risk of cardiometabolic disorders in lean non-alcoholic fatty liver disease: A longitudinal study. Am. J. Prev. Cardiol..

[42] Zhu X., Huang Q., Ma S., Chen L., Wu Q., Wu L., Ma H., Li X., Li Q., Aleteng Q. (2023). Presence of sarcopenia identifies a special group of lean NAFLD in middle-aged and older people. Hepatol. Int..

[43] Zhang X., He Z., Si Q., Hu X., Yang L., Gu X., Du L., Wang L., Pan L., Li Y. (2022). The Association of Sarcopenia and Visceral Obesity with Lean Nonalcoholic Fatty Liver Disease in Chinese Patients with Type 2 Diabetes Mellitus. J. Diabetes Res..

[44] Lan Y., Lu Y., Li J., Hu S., Chen S., Wang Y., Yuan X., Liu H., Wang X., Wu S. (2022). Outcomes of subjects who are lean, overweight or obese with nonalcoholic fatty liver disease: A cohort study in China. Hepatol. Commun..

[45] Nabi O., Lapidus N., Boursier J., de Ledinghen V., Petit J.M., Kab S., Renuy A., Zins M., Lacombe K., Serfaty L. (2023). Lean individuals with NAFLD have more severe liver disease and poorer clinical outcomes (NASH-CO Study). Hepatology.

[46] Rastogi A., Rath I., Varadarajan A., Ramakrishna G., Bihari C., Maiwall R. (2022). Non-alcoholic fatty liver disease (NAFLD) in lean individuals—Single centre large cohort clinicopathologic and immunophenotypic study. Pathol. Res. Pract..

[47] Kim Y., Han E., Lee J.S., Lee H.W., Kim B.K., Kim M.K., Kim H.S., Park J.Y., Kim D.Y., Ahn S.H. (2022). Cardiovascular Risk Is Elevated in Lean Subjects with Nonalcoholic Fatty Liver Disease. Gut Liver.

[48] Hagstrom H., Nasr P., Ekstedt M., Hammar U., Stal P., Hultcrantz R., Kechagias S. (2018). Risk for development of severe liver disease in lean patients with nonalcoholic fatty liver disease: A long-term follow-up study. Hepatol. Commun..

[49] Lu C.W., Yang K.C., Chi Y.C., Wu T.Y., Chiang C.H., Chang H.H., Huang K.C., Yang W.S. (2023). Adiponectin-leptin ratio for the early detection of lean non-alcoholic fatty liver disease independent of insulin resistance. Ann. Med..

[50] Chahal D., Sharma D., Keshavarzi S., Arisar F.A.Q., Patel K., Xu W., Bhat M. (2022). Distinctive clinical and genetic features of lean vs overweight fatty liver disease using the UK Biobank. Hepatol. Int..

[51] Weinberg E.M., Trinh H.N., Firpi R.J., Bhamidimarri K.R., Klein S., Durlam J., Watkins S., Reddy K.R., Weiss M., Zink R.C. (2021). Lean Americans With Nonalcoholic Fatty Liver Disease Have Lower Rates of Cirrhosis and Comorbid Diseases. Clin. Gastroenterol. Hepatol..

[52] Cruz A.C.D., Bugianesi E., George J., Day C.P., Liaquat H.B., Charatcharoenwitthaya P., Mills P.R., Dam-Larsen S., Bjornsson E.S., Haflidadottir S. (2014). 379 Characteristics and Long-Term Prognosis of Lean Patients with Nonalcoholic Fatty Liver Disease. Gastroenterology.

[53] Freeman A.M., Acevedo L.A., Pennings N. (2023). Insulin Resistance. StatPearls.

[54] Borai A., Livingstone C., Abdelaal F., Bawazeer A., Keti V., Ferns G. (2011). The relationship between glycosylated haemoglobin (HbA1c) and measures of insulin resistance across a range of glucose tolerance. Scand J. Clin. Lab. Investig..

[55] Rochlani Y., Pothineni N.V., Kovelamudi S., Mehta J.L. (2017). Metabolic syndrome: Pathophysiology, management, and modulation by natural compounds. Ther. Adv. Cardiovasc. Dis..

[56] Almomani A., Kumar P., Onwuzo S., Boustany A., Krishtopaytis E., Hitawala A., Alshaikh D., Albakri A., Hussein L., Hussein E. (2023). Epidemiology and prevalence of lean nonalcoholic fatty liver disease and associated cirrhosis, hepatocellular carcinoma, and cardiovascular outcomes in the United States: A population-based study and review of literature. J. Gastroenterol. Hepatol..

[57] Ahmed O.T., Gidener T., Mara K.C., Larson J.J., Therneau T.M., Allen A.M. (2022). Natural History of Nonalcoholic Fatty Liver Disease With Normal Body Mass Index: A Population-Based Study. Clin. Gastroenterol. Hepatol..

[58] Eslam M., Ahmed A., Despres J.P., Jha V., Halford J.C.G., Wei Chieh J.T., Harris D.C.H., Nangaku M., Colagiuri S., Targher G. (2021). Incorporating fatty liver disease in multidisciplinary care and novel clinical trial designs for patients with metabolic diseases. Lancet Gastroenterol. Hepatol..

[59] Long M.T., Noureddin M., Lim J.K. (2022). AGA Clinical Practice Update: Diagnosis and Management of Nonalcoholic Fatty Liver Disease in Lean Individuals: Expert Review. Gastroenterology.

[60] Rinella M.E., Lazarus J.V., Ratziu V., Francque S.M., Sanyal A.J., Kanwal F., Romero D., Abdelmalek M.F., Anstee Q.M., Arab J.P. (2023). A multi-society Delphi consensus statement on new fatty liver disease nomenclature. J. Hepatol..

[61] Song S.J., Lai J.C., Wong G.L., Wong V.W., Yip T.C. (2023). Can we use old NAFLD data under the new MASLD definition?. J. Hepatol..

[62] Geh D., Manas D.M., Reeves H.L. (2021). Hepatocellular carcinoma in non-alcoholic fatty liver disease-a review of an emerging challenge facing clinicians. Hepatobiliary Surg. Nutr..

[63] Steffl M., Bohannon R.W., Sontakova L., Tufano J.J., Shiells K., Holmerova I. (2017). Relationship between sarcopenia and physical activity in older people: A systematic review and meta-analysis. Clin. Interv. Aging.

[64] Meier N.F., Lee D.C. (2020). Physical activity and sarcopenia in older adults. Aging Clin. Exp. Res..

[65] Premkumar M., Anand A.C. (2021). Lean Fatty Liver Disease: Through Thick and Thin. J. Clin. Exp. Hepatol..

[66] Choi H.M., Doss H.M., Kim K.S. (2020). Multifaceted Physiological Roles of Adiponectin in Inflammation and Diseases. Int. J. Mol. Sci..

[67] Abella V., Scotece M., Conde J., Pino J., Gonzalez-Gay M.A., Gomez-Reino J.J., Mera A., Lago F., Gomez R., Gualillo O. (2017). Leptin in the interplay of inflammation, metabolism and immune system disorders. Nat. Rev. Rheumatol..

[68] Fruhbeck G., Catalan V., Rodriguez A., Ramirez B., Becerril S., Salvador J., Colina I., Gomez-Ambrosi J. (2019). Adiponectin-leptin Ratio is a Functional Biomarker of Adipose Tissue Inflammation. Nutrients.

[69] Duarte S.M.B., Stefano J.T., Miele L., Ponziani F.R., Souza-Basqueira M., Okada L., de Barros Costa F.G., Toda K., Mazo D.F.C., Sabino E.C. (2018). Gut microbiome composition in lean patients with NASH is associated with liver damage independent of caloric intake: A prospective pilot study. Nutr. Metab. Cardiovasc. Dis..

[70] Xue L., Deng Z., Luo W., He X., Chen Y. (2022). Effect of Fecal Microbiota Transplantation on Non-Alcoholic Fatty Liver Disease: A Randomized Clinical Trial. Front. Cell Infect. Microbiol..

[71] Park H., Yoon E.L., Cho S., Jun D.W., Nah E.H. (2022). Diabetes is the strongest risk factor of hepatic fibrosis in lean patients with non-alcoholic fatty liver disease. Gut.

[72] Vilarinho S., Ajmera V., Zheng M., Loomba R. (2021). Emerging Role of Genomic Analysis in Clinical Evaluation of Lean Individuals With NAFLD. Hepatology.

[73] Wong V.W., Wong G.L., Chan R.S., Shu S.S., Cheung B.H., Li L.S., Chim A.M., Chan C.K., Leung J.K., Chu W.C. (2018). Beneficial effects of lifestyle intervention in non-obese patients with non-alcoholic fatty liver disease. J. Hepatol..

[74] Julien J., Ayer T., Tapper E.B., Barbosa C., Dowd W.N., Chhatwal J. (2022). Effect of increased alcohol consumption during COVID-19 pandemic on alcohol-associated liver disease: A modeling study. Hepatology.

[75] Danpanichkul P., Ng C.H., Muthiah M., Suparan K., Hao Tan D.J., Duangsonk K., Sukphutanan B., Kongarin S., Harinwan N., Panpradist N. (2023). From Shadows to Spotlight: Exploring the Escalating Burden of Alcohol-Associated Liver Disease and Alcohol Use Disorder in Young Women. Am. J. Gastroenterol..

[76] Odriozola A., Santos-Laso A., Del Barrio M., Cabezas J., Iruzubieta P., Arias-Loste M.T., Rivas C., Duque J.C.R., Anton A., Fabrega E. (2023). Fatty Liver Disease, Metabolism and Alcohol Interplay: A Comprehensive Review. Int. J. Mol Sci.

[77] Liangpunsakul S., Lai X., Ross R.A., Yu Z., Modlik E., Westerhold C., Heathers L., Paul R., O’Connor S., Crabb D.W. (2015). Novel serum biomarkers for detection of excessive alcohol use. Alcohol. Clin. Exp. Res..

[78] Rinella M.E., Neuschwander-Tetri B.A., Siddiqui M.S., Abdelmalek M.F., Caldwell S., Barb D., Kleiner D.E., Loomba R. (2023). AASLD Practice Guidance on the clinical assessment and management of nonalcoholic fatty liver disease. Hepatology.

[79] Souza M., Diaz I., Barchetta I., Mantovani A. (2023). Gastrointestinal cancers in lean individuals with non-alcoholic fatty liver disease: A systematic review and meta-analysis. Liver Int..

[80] Ramai D., Singh J., Lester J., Khan S.R., Chandan S., Tartaglia N., Ambrosi A., Serviddio G., Facciorusso A. (2021). Systematic review with meta-analysis: Bariatric surgery reduces the incidence of hepatocellular carcinoma. Aliment. Pharmacol. Ther..

[81] Stine J.G., DiJoseph K., Pattison Z., Harrington A., Chinchilli V.M., Schmitz K.H., Loomba R. (2023). Exercise Training Is Associated With Treatment Response in Liver Fat Content by Magnetic Resonance Imaging Independent of Clinically Significant Body Weight Loss in Patients With Nonalcoholic Fatty Liver Disease: A Systematic Review and Meta-Analysis. Am. J. Gastroenterol..

